# Prevention and improved management of serious neurological adverse events during praziquantel-based mass drug administration in a *Taenia solium* endemic area: Experiences from Madagascar

**DOI:** 10.1371/journal.pntd.0012590

**Published:** 2025-04-17

**Authors:** José Alphonse Nely, Noromanana Sylvia Ramiandrasoa, Diana Edithe Andria-Mananjara, Glenn Edosoa, Patricia Rasoamihanta Martin, Bernadette Abela, Meritxell Donadeu, Agnès Fleury

**Affiliations:** 1 Direction de lutte contre les maladies transmisibles, Ministry of Health of Madagascar, ex-IHS Analakely, Antananarivo, Madagascar; 2 Andrambato Itaosy, Antananarivo, Madagascar; 3 National Center for Applied Research on Rural Development (FOFIFA), Antananarivo, Madagascar; 4 World Health Organization Madagascar Office, Antananarivo, Madagascar; 5 Department of the Control of Neglected Tropical Diseases, World Health Organization, Geneva, Switzerland; 6 Department of Biosciences, Melbourne Veterinary School, University of Melbourne, Werribee, Victoria, Australia; 7 Initiative for Neglected Animal Diseases (INAND), Pretoria, South Africa; 8 Departamento de Medicina Genómica y Toxicología Ambiental, Instituto de Investigaciones Biomédicas, Universidad Nacional Autónoma de México, Ciudad de México, México; 9 Clínica de neurocisticercosis, Instituto Nacional de Neurología y Neurocirugía, Ciudad de México, México; University of Washington, UNITED STATES OF AMERICA

## Abstract

Mass drug administration (MDA) programs involving praziquantel are used in public health programs to control diseases such as schistosomiasis, taeniasis caused by *Taenia solium*, opisthorchiasis and clonorchiasis. Praziquantel is a systemically distributed anthelmintic drug also used to treat neurocysticercosis (NCC) caused by the larval stages of *T. solium* in the central nervous system. The doses of praziquantel used in MDA are low compared to those used for the treatment of NCC, but in people with latent NCC (without symptoms or signs), there is a potential risk of neurological adverse events (AE) due to the development of inflammation around the cysts following administration. In Madagascar two large MDA campaigns aimed at *T. solium* were conducted using praziquantel in the Vakinankaratra region. Prior to the first MDA campaign, we implemented a program designed to minimize the occurrence of neurological AE and improve their management, which included training of health agents and community workers as well as health centres staff, population awareness, post-MDA active and passive surveillance and the supply of basic medicines to health centres. This program was repeated for the second MDA campaign. A total of 117,216 and 163,089 people were treated during the first and second MDA campaign respectively, with 10 participants experiencing serious AE, which were successfully managed. The beneficial results from our program in Madagascar can help other programs and countries using MDA with praziquantel in *T. solium* endemic areas to improve the safety of these campaigns.

## Author summary

Mass drug administration (MDA) with praziquantel is an important tool to combat several diseases, including taeniasis caused by the adult form of *Taenia solium.* The larvae of *T. solium* frequently infects the central nervous system of humans, causing neurocysticercosis. Since praziquantel is active not only on the adult form of the parasite, but also on its larval form, there are risks of neurological adverse events (seizures or symptoms of intracranial hypertension) if individuals who are treated for possible taeniasis, have neurocysticercosis. These adverse events are infrequent but can be life-threatening if not managed promptly in a correct manner. In Madagascar we set up a program to minimise those neurological adverse events, quickly identify them if they occurred, and to manage them appropriately. Following the implementation of this program, two MDA campaigns were carried out. A total of 280,305 people received treatment, with 10 experiencing neurological side effects. All cases were well managed, and all are now in good health. Here we describe our experiences, as we believe they can be useful to other programs using praziquantel in *T. solium* endemic areas.

## Introduction

Mass drug administration (MDA) programs which involve the administration of praziquantel are a valuable public health tool used to control various neglected parasitic diseases in endemic communities. Examples include schistosomiasis, taeniasis caused by *Taenia solium*, opisthorchiasis and clonorchiasis. The largest of these programs is that for schistosomiasis, which since 2006 had reached over 100 million people by 2019 [1], and in 2021 was required in 51 countries for a total of 251.4 million people [2].

Concern has been raised about the use of praziquantel in MDA in people who may be infected with the viable larval stages of *T. solium* in the central nervous system (neurocysticercosis, NCC) [3]. This is because praziquantel is active on the larval form of *T. solium* present in the brain due to its capacity to cross the blood brain barrier [4–6], as well as being effective on adult *T. solium* parasites due to its local intestinal action. Praziquantel is thus one of the two anthelminthic drugs used to treat neurocysticercosis, after the diagnosis is confirmed by neuroimaging studies. Although NCC is a major cause of seizures and epilepsy in the communities in which the parasite is present, NCC can remain asymptomatic for as long as 30 years [7]. While conducting MDA with praziquantel for taeniasis or other diseases in *T. solium* endemic areas, people with asymptomatic NCC may receive praziquantel. In this case, although the doses of praziquantel used for MDA (taeniasis: a single dose of 10mg/kg; schistosomiasis and small liver fluke programs: a single dose of 40mg/kg) are much lower than those used for the treatment of NCC (50mg/kg/day for 10–14 days), it can nevertheless be sufficient to reach the brain causing inflammation of the cysts and neurological symptoms [6]. This situation seems to be infrequent, as millions of people have received MDA in *T. solium* endemic areas [2,8,9] and only few neurological adverse events (AE) linked to NCC have been reported. These were mainly epileptic seizures, including status epilepticus, and intracranial hypertension [10] which may be life-threatening. Of course, under-reporting cannot be totally ruled out, not least because of the persistent stigma attached to epilepsy in many endemic countries [11,12]. While these concerns have been raised in various fora [3,13], and very good information is available on the safety of administering medicines for neglected tropical diseases [14], there is limited information or detailed policies aimed to avoid these serious AE and to outline best practice for managing neurological AE should they occur.

In Madagascar, MDA with praziquantel has been used widely as part of the schistosomiasis control program and also in a large-scale pilot for taeniasis conducted between 2015 and 2017 [15]. During December 2021, as part of a large One Health program for the control of *T. solium* in the Vakinankaratra region of Madagascar, the Ministry of Health of Madagascar intended to implement an MDA campaign with praziquantel. Tragically, some days before the campaign was due, a boy in a different but nearby area died following treatment with praziquantel as part of the MDA program for schistosomiasis. Due to the characteristics of the clinical presentation, it was considered likely, although not confirmed, that the event was caused by the development of an obstructive hydrocephalus linked to a latent NCC. As a result, the Ministry of Health ceased all MDA in the country until further prevention and management activities were put in place. Partners in the planned *T. solium* project, including the Ministry of Health of Madagascar and the World Health Organization (WHO), as well as the clinicians and consultant neurologist, worked together to develop and implement a plan to prevent and minimise neurological AE when using praziquantel. In this paper we describe the plan to prevent and minimise neurological AE, and its implementation and results during two MDAs aimed at taeniasis in 2022 and 2024.

## Methods

### Ethics statement

Intervention and assessment protocols were designed and implemented in compliance with ethical approvals for the study granted by the Ethics Committee for Biomedical Research of the Ministry for Public Health Madagascar No.88-MSANP/SG/AMM/CERBM.

Diagnostic procedures were undertaken by the patient’s treating physician. Written informed consent was obtained from the parent of the child whose CT images are included in this article.

### Plan and activities to minimise neurological AE

**Fig 1:**
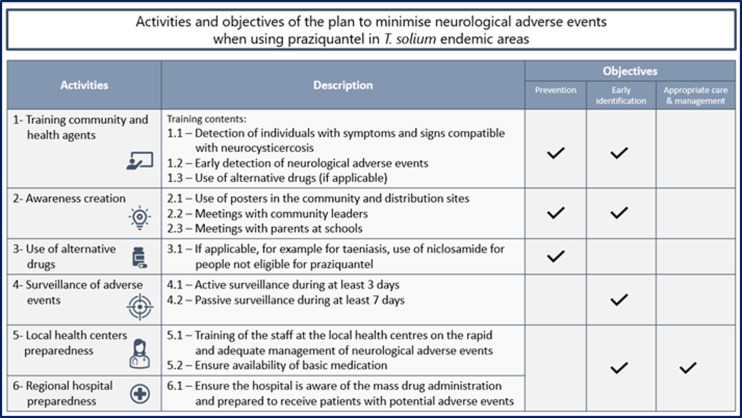
Activities and objectives of the plan.

**Fig 2:**
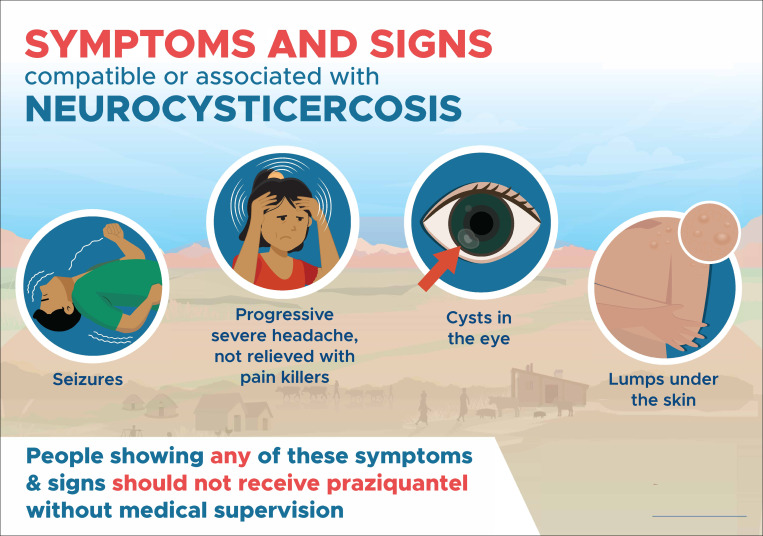
English version of the poster used for training and awareness on symptoms and signs compatible with neurocysticercosis. Source: World Health Organization [17]: https://www.who.int/publications/m/item/WHO-UCN-NTD-VVE-2022.2.

**Fig 3:**
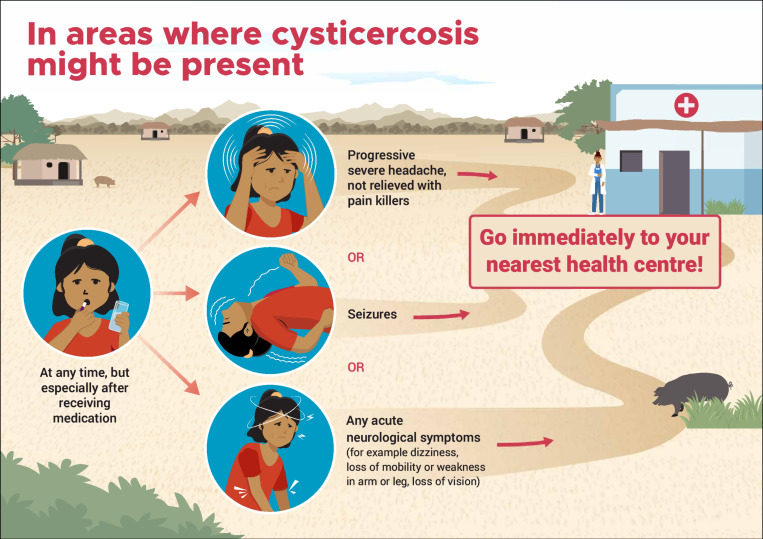
English version of the poster used for training and awareness on early detection of neurological AE. Source: World Health Organization [18]: https://www.who.int/publications/m/item/WHO-UCN-NTD-VVE-2022.1.

Before starting the MDA with praziquantel, a plan was developed to reduce the risk of neurological AE related to NCC, and to improve their management in case of occurrence with 3 objectives:

*Objective 1: Prevention of neurological AE by detecting individuals with symptoms and signs compatible with NCC*. This is essential as these individuals have a clear contraindication to receive praziquantel during the MDA [3]. Praziquantel can generate inflammation around the cerebral cysts and worsen the neurological signs [6]. To avoid this occurrence the individuals with symptoms compatible with NCC (epileptic seizures, progressive intense headaches unresponsive to conventional pain killers, focal deficits, decreased vision, impaired speech, and ataxia) should not receive praziquantel. In addition, as the presence of cysts outside the central nervous system may be associated with NCC, people with ocular or subcutaneous cysticercosis should not receive praziquantel either [16]. In these cases, niclosamide can be provided as an alternative to praziquantel for taeniasis, as per the WHO guidelines for taeniasis [3]. The advantage of niclosamide is that it is not absorbed from the gut and does not have a systemic effect.
*Objective 2: Early detection of neurological AE.* This is critical because the immediate attention of patients is essential to reduce morbidity and mortality.
*Objective 3: Rapid and adequate management of neurological AE.* All patients should receive adequate care with the right medication.
To achieve these objectives, six different activities were included in the plan (Fig 1):

1. **Training of community health agents (CHA) and health agents (HA):** This activity was aimed at supporting objectives 1 and 2. In Madagascar CHA are part of the community, there are typically two per fokontany (the smaller administrative unit), and usually they provide the MDA medication. HA are staff of the Ministry of Health and act as supervisors. We used the training of the trainers’ cascade to enable CHA and HA to:

- Distinguish symptoms and signs compatible with NCC, to identify people not eligible for MDA with praziquantel. The WHO produced a document and a poster (Fig 2) to assist with the training [16,17].
- Identify rapidly neurological AE. It was emphasized that NCC is a pre-existing condition in a very small proportion of the participants, and it is not caused by the medication. This clarified that neurological AEs could not occur in all participants, as well as avoiding unwarranted fears associated with MDA. WHO and the Ministry of Health produced a poster (Fig 3) [18] to assist with the training.
- Correctly administer niclosamide. People not eligible for praziquantel would be offered niclosamide. It is very important that niclosamide is properly chewed to ensure its efficacy. This was the first time that niclosamide was used in Madagascar so special training was provided, including dose calculation (adults 2g; children 10–35 Kg 1g).

1. **Awareness creation**: This activity was conducted to support objectives 1 and 2. Several strategies were used to create awareness in the community of the symptoms and signs compatible with NCC, as well as the early identification of neurological AE.

- The posters mentioned above (Figs 2 and 3) were used in each location where the drugs were administered, and the community agents worked door to door.
- Meetings were conducted with the village leaders and with parents at schools.

1. **Delivery of alternative drugs:** As part of the prevention activities, people who were identified with symptoms and signs compatible with NCC were offered niclosamide instead.
2. **Surveillance**: This activity was to support objective 2 and in accordance with the Pan American Health Organization recommendations [3]. Active surveillance was conducted during and after the MDA campaigns. The MDA campaigns lasted each 8 days; for the first five days the CHA delivered praziquantel and identified people non eligible to receive it; the following three days, they delivered niclosamide to the people who had been identified as non-eligible for praziquantel. During all this time, they were active in the community seeking to identify any potential neurological AE by going door to door. During the following 7 days the CHA were still present in the community, attending any putative AE.
3. **Local health centres preparedness**: These efforts were aimed at objective 3, and included two main components:

- Training of health staff in the local health centres. They were trained in clinical and therapeutical aspects of procedures, drugs, and case management according to the available facilities [19], when to refer the patient to hospital, and on the messages to be communicated to the patient and the family, such as the medication didn’t cause the disease, but ‘revealed it’.
- Provision of basic medication to the health centres. Some of the local health centres did not have the appropriate medication or supplies to deal with neurological emergencies so it was provided. The suggested drugs to be made available at all health centres are diazepam or midazolam as antiseizure medications, and oral/IM corticosteroids to manage inflammation [19]. At health centres with intravenous capability, they should also include valproic acid or phenytoin (ideally both) and IV corticosteroids [20]. In several endemic countries phenobarbital is the antiseizure medication mostly used. Its utilization should be done with precaution due to the risk of respiratory depression.

1. **Regional hospital preparedness:** This action was to support objective 3. The regional hospital was made aware of the campaign, so that they were prepared to deal with referrals.

### Population included and study site

The target population were children over 5 years of age and adults of 9 communes of Betafo and Mandoto districts in the Vakinankaratra Region of Madagascar. This area comprises 420 villages over 84 fokontany, approximately 220,000 inhabitants, and 21 primary health centres and 3 regional hospitals (1 public and 2 private hospitals) are in operation. The region is known for its high prevalence of neurocysticercosis [21] and porcine cysticercosis [22,23].

### Mass drug administration

Two MDAs were conduct, one in August 2022 and the other one in February 2024. The MDAs were implemented by the Ministry of Public Health of Madagascar using the protocols in place for the schistosomiasis program and used in the previous pilot project for taeniasis [15]. The MDAs were organized in a hybrid way, mainly in households (i.e., door-to-door), but also to work in synergy with the MDA program on schistosomiasis, in schools and health centres by taking advantage of the activities planned within this framework. In brief, the CHA provided the treatment and HA of the Ministry of Public Health acted as supervisors. During the first MDA in August 2022, children were targeted for taeniasis and schistosomiasis and received a dose of praziquantel of 40mg/kg while the adults received the taeniasis dose of praziquantel of 10mg/kg. During the second MDA in February 2024, in Mandoto children and adults received praziquantel at 10mg/kg (albeit few children received 40mg/kg), while in Betafo some children received praziquantel at 40mg/kg to target taeniasis and schistosomiasis while the rest of children and the adults received praziquantel at 10 mg/kg. Additionally, children in Betafo also received mebendazole (500mg) for the control of soil-transmitted helminthiases in both MDAs.

## Results

The six activities aimed at the prevention, early identification and management of neurological AE were first implemented during July 2022. The training on the prevention and early identification of neurological AE using the training of the trainers’ cascade model included 13 HA, and 255 CHA. Awareness campaigns for the MDA were conducted from the end of July until the beginning of August, and also included ‘town criers’ and traditional Malagasy theatre ‘Hira Gasy’. Parallel activities were conducted to create awareness on the symptoms and signs compatible with NCC in the community, including discussions with parents of school children. During the MDA, when the CHA delivered praziquantel (door-to-door, or in health centres and schools, which, when possible, also exhibited the posters from Figs 2 and 3), they were asking questions to identify people with symptoms and signs compatible with NCC as per the training, and people not eligible to receive praziquantel were later given niclosamide. The CHA are members of the community, and they remained ‘visible’ and accessible to the community during the surveillance period. Similar activities were conducted before the second MDA in February 2024.

At the time of each MDA, the local health centres were ready to receive any potential neurological AE, and the regional hospital staff were aware of the MDA process and prepared to receive any patients.

In August 2022, a total of 117,216 individuals were treated over a total of 187,490 eligible population. A total of 42,438 children and 74,163 adults received praziquantel, and 110 children and 505 adults (0.52% of the treated population) received niclosamide as they presented symptoms compatible with NCC/non-neurological cysticercosis. In the second MDA, 163,089 individuals were treated over a total of 196,791 eligible population. A total of 58,773 children and 103,333 adults received praziquantel, and 235 children and 748 adults received niclosamide (0.60% of the treated population) (Table 1). The low coverage achieved in 2022, is due to the concerns after the fatality that occurred in a nearby area during the MDA campaign of December 2021. The higher coverage in 2024, reflects the increased confidence of the population.

**Table 1:** Number of children and adults that received praziquantel or niclosamide during the MDAs.

Year	Drug	Mandoto		Betafo		Total per drug	Total per year	Eligible population (coverage %)
		Children	Adults	Children	Adults			
2022	Praziquantel	23,065	43,322	19,373	30,841	116,601	117,216	187,490 (62.5%)
	Niclosamide	81	303	29	202	615		
2024	Praziquantel	29,747	51,571	29,026	51,762	162,106	163,089	196,791 (82.9%)
	Niclosamide	92	314	143	434	983		

**Fig 4:**
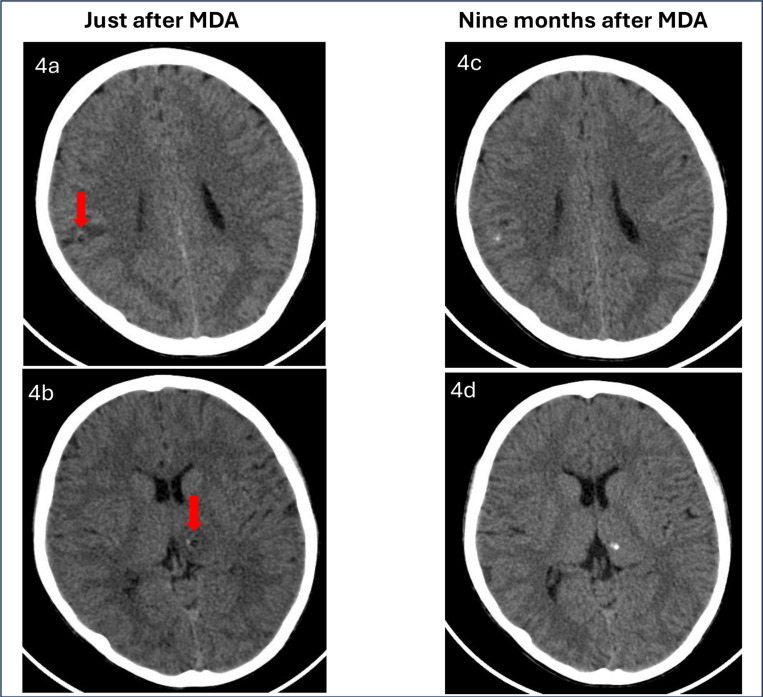
Computed tomography (CT) scan images of the boy who presented seizures after the MDA in 2022. 4a: colloidal stage, 4b: vesicular stage, 4c, d: calcifications.

In the first MDA, August 2022, one serious neurological AE was identified, during the first day of the campaign. A 13-year-old boy from Mandoto, in the evening of the first day of MDA presented headache, which became stronger during the night; the next day, he vomited and had seizures. The CHA brought the child and his parents to the Health Center in Ankazomiriotra where he received appropriate care (anti-seizure medication and painkillers, as well as a check-up of his vital signs and general condition). A computed tomography (CT) scan done a few days after the event confirmed the presence of three parenchymal NCC lesions (Fig 4): one with clear inflammatory reaction around (Fig 4a-colloidal stage), one viable with discrete inflammatory reaction around (Fig 4b–vesicular stage), and one calcified parasite.

Under the guidance of medical experts, it was decided not to give anthelminthic treatment as all the cysts showed clear signs of degeneration. Anti-seizure medication (valproic acid) was continued, there was no recurrence of seizures, and the child is currently well, having a normal life. A CT scan made nine months after, showed calcification of the vesicular and the colloidal cysts (Fig 4c and 4d). In the subsequent MDA in 2024, the boy received niclosamide.

In the second MDA, in February 2024, six people reported epileptic seizures between half an hour to 6 days after the administration of the praziquantel (they all reported it was the first time they had experienced them), and three children presented intense headache (Table 2). Of them, six had received praziquantel 10mg/kg, and three praziquantel 40 mg/kg (Table 2). Adequate treatment (anti-seizure medication and painkillers) was administered, and in all the cases the symptoms were controlled without recidivism. Contrast enhanced CT scans were conducted 3 months after the MDA to 8 individuals; the parents of one of the children with epileptic seizures did not consent. In three of them (2 children with seizures and one with intense headache), calcifications very suggestive of NCC were observed, associated in one of the patients with an inflammatory granuloma. The cause of the granuloma was unclear, as it could be due to NCC or tuberculosis. The affected boy was in good general health, his chest X-ray was normal, he had had no contact with people with tuberculosis, and the PCR on cerebrospinal fluid was negative for tuberculosis, all of which argue in favour of a diagnosis of NCC. At the time of writing this paper in February 2025 the boy is still receiving anti-seizure medication (phenobarbital) as when it was attempted to stop some months previously, the seizures reappeared.

**Table 2:** Details of the persons presenting serious adverse events during the Feb 2024 MDA. Children in Betafo also received mebendazole (500mg) for the control of soil-transmitted helminthiases.

Age	Sex	District	Symptoms reported by community health agents	Time onset after MDA	Praziquantel dose	Scan results (3 months after MDA)
Presenting seizures						
8	M	Mandoto	Seizures	½ hour	10mg/kg	Normal
5	M	Mandoto	Seizures, headache	½ hour	10mg/kg	NCC calcification on the right basal nuclei. Partially calcified granuloma with left parietal peri-lesional oedema and contrast enhancement. NCC vs tuberculosis.
12	F	Mandoto	Seizures	72 hours	40mg/kg	Did not consent to scan
11	M	Betafo	Seizures, headache, nausea	1 day	10mg/kg	Normal
25	F	Mandoto	Seizures, headache	2 days	10mg/kg	Normal
10	M	Mandoto	Seizures	6 days	10mg/kg	Two NCC calcifications, one parietal and the other occipital, both on the left. No contrast enhancement.
Presenting intense headache						
11	F	Betafo	Headache, dizziness, vomits	2h	40mg/kg	Normal
12	F	Betafo	Intense headache, acute abdominal pain	2h	40mg/kg	Normal
9	M	Betafo	Headache, vomits, asthenia	7h	10mg/kg	Right frontal NCC calcification.

## Discussion

The program described here, which sought to minimize the potential for AE following MDA with praziquantel and to identify and manage AE, provided the government of Madagascar with sufficient confidence that the country’s ban on MDAs was lifted, and we were permitted to proceed. More than 280,000 people were treated during two MDAs and 10 serious AE were recorded and treated, all of whom are currently in good health.

A total of 1,598 people were identified with signs compatible with possible NCC and these people were treated with niclosamide, among whom there were no AE. It is not possible to estimate how many, if any, AE may have been avoided among these people who were not treated with praziquantel. The re-direction of suspect NCC cases to take niclosamide rather than praziquantel, could potentially have avoided more serious sequalae, or even deaths.

This was the first time that an MDA for taeniasis considered a participant’s neurological symptoms and signs compatible with NCC prior to treatment, leading to a discrimination between those offered niclosamide rather than praziquantel. Praziquantel is preferred for treatment of taeniasis because it is considered to be almost always effective [24], whereas a single treatment with niclosamide leaves 30% or more of taeniasis carriers remaining in the community as sources of new cases of neurocysticercosis [25]. With this in mind, we believe that our strategy has been the most effective in avoiding serious AEs without losing the preventive effect targeted by the MDA. In the future, other potential developments such as a reliable point-of-care tests to detect active NCC, might help to improve and refine this strategy.

The percentage of the population that were selected to receive niclosamide, was similar during the two MDA programs (0.58% of all treatments, 0.52% during the first MDA, and 0.6% during the second MDA). This is useful information for planning the niclosamide needs for future campaigns. While it is not possible to know the effectiveness of this measure, having this as an option is of importance for future MDAs.

Although MDA with praziquantel is safe for the vast majority of individuals, serious neurological AE can occur when asymptomatic *T. solium* cysts are located in the central nervous system. Due to the absence of symptoms, the exact prevalence of asymptomatic NCC is unknown, but is probably high given the latency of several years between infection and symptoms [7,26–28]. As endemicity of *T. solium* is clearly linked to poverty (the *T. solium* life cycle requires traditional pig farming with roaming pigs, open defecation and inadequate sanitation), health systems in endemic areas are often precarious. Although MDAs have usually been implemented with all the necessary precautions and information, a standardized program to reduce the risk of neurological AE and improve their management in *T. solium* endemic areas was lacking.

One of the strengths of the program we developed, is its potential applicability to other endemic regions. The program included specific posters that were designed and distributed to clearly present the contraindications of praziquantel, as well as the signs/symptoms that should lead patients to be transferred to a health centre. These posters were used both during training sessions and for community awareness-raising. They have been translated into several languages by WHO and made available to organizations/governments interested in implementing MDA with praziquantel in *T. solium* endemic areas. On the other hand, basic medications were supplied to the health centres, enabling adequate management of any neurological AE, which were mainly epileptic seizures and intracranial hypertension. The main drugs were corticoids and various anticonvulsants, and health centre staff were trained in their use.

Nine CT scans were carried out in patients with SAEs (one in 2022 and 8 in 2024); 4 of these (44.4%) showed images compatible with NCC. All the cysts were calcified, with the exception of the patient diagnosed in 2022 (Fig 4), and of one of the patients diagnosed in 2024 who, in addition to one calcified lesion, presented an inflammatory granuloma, highly suspicious of NCC. It is relevant to note that in 2022 CT scan was done quickly after the seizure in the local CT scan device at the regional hospital. Contrarily, and unfortunately, in 2024 due to the local CT scan breaking, the patients had to be taken to the capital for the CT scans, that were done 3 months after the symptoms. These three CT scans evidenced calcified lesions in all, associated in one of the patients with an inflammatory granuloma. The higher proportion of calcified NCC cysts when CT scans, as in 2024, were performed longer after symptoms is consistent with the evolution of NCC cysts. Indeed, it is reasonable to assume that the symptoms were due to inflammation around the NCC cysts after praziquantel administration. Then, the process of cyst degeneration probably lasted a few weeks, ending with their calcification, which was the lesion visible on the CT scans.

It is interesting to note that the age range of the patients with neurological AE was 5–25 years of age, and the age range for the patients in which NCC was confirmed was 5–13 years of age. No adults were identified. The difference in frequency of neurological AEs between adults and children could be explained by the increased susceptibility of children, compared to adults, to develop seizures [29] as well as to the known increase in children’s reactivity against cysticerci, demonstrated by the high frequency of degenerating cysts in this age group [30].

Of the 4 NCC confirmed cases, 3 were from Mandoto (1 during the first MDA, and 2 during the second MDA), and 1 from Betafo (the area in which mebendazole was also given to children). There is very limited information about the safe use of praziquantel simultaneously with mebendazole in *T. solium* endemic areas, and we hope the information provided here, contributes to build that knowledge.

The presence of *T. solium* in Madagascar is well known [21,22,31], and it has one of the highest rates of porcine cysticercosis infection ever reported [23]. However, as CT scanning remains unaffordable and inaccessible for most Malagasy patients, information regarding neurocysticercosis cases is infrequent. Unfortunately, we are not in a position to know how many AE were prevented thanks to the implementation of our program. But the results of the CT scans (four out of nine (44.4%) revealed the presence of NCC) confirmed the high transmission of *T. solium* in the region and suggest that the number could have been much higher if the program had not been implemented. The high *T. solium* endemicity in the area, and the awareness and sensitisation campaigns that encouraged people to come forward with adverse events, might help to explain the numbers of AE identified during the MDAs campaigns.

Our experience demonstrated the importance to have a plan, before MDA with praziquantel is implemented in an area likely to be endemic for *T. solium*, for the confirmation and follow-up of any potential NCC cases identified. CT-scans are necessary in these cases and are often not readily available in regions where MDA is performed. This must be taken into account in MDA planning, not least for the cost it may represent.

An additional benefit of the MDAs described here was that during its implementation, several people who suffered epilepsy but were not receiving antiseizure medication approached the HA and enquired about treatment. We believe that the awareness and the sensitisation campaigns assisted in encouraging people to request medical assistance to manage their condition. Indeed, epilepsy is still stigmatized in many countries, and the program was able to contribute to a better perception of what this illness really is.

During our MDA with praziquantel not all cases of neurological AE could be avoided, nevertheless we ensured that at the best of our knowledge and capabilities, those were minimised, and the necessary actions were taken to provide adequate care and minimise fatalities. MDA with praziquantel is a key tool to control several neglected diseases specially in children, so it is important to continue its implementation for the benefits for many while minimizing the risks [3]. We hope that the policies and procedures developed by this group, and the lessons from Madagascar can help other programs and countries using MDA with praziquantel in *T. solium* endemic areas, and that affected communities will benefit.

## References

[1] World Health Organization. 34th meeting of the International Task Force for Disease Eradication, 19–20 September 2022. Wkly Epidemiol Rec. 2023;4(98):41–50. https://www.who.int/publications/i/item/who-wer9804-41-50

[2] World Health Organization. Schistosomiasis and soil-transmitted helminthiases: progress report, 2021. Wkly Epidemiol Rec. 2022;48(97):621–32. https://www.who.int/publications/i/item/who-wer9748-621-632

[3] Pan American Health Organization. Guideline for preventive chemotherapy for the control of *Taenia solium* taeniasis. Washington, D.C: Pan American Health Organization; 2021. https://www.who.int/publications/i/item/9789275123720 36473068

[4] Sotelo J, Jung H. Pharmacokinetic optimisation of the treatment of neurocysticercosis. Clin Pharmacokinetics. 1998;34(6):503–15. doi: 10.2165/00003088-199834060-00006 9646011

[5] World Health Organization. WHO model prescribing information: drugs used in parasitic diseases. 2nd ed. Geneva: World Health Organization; 1995. p. 146. https://iris.who.int/handle/10665/41765

[6] Hamamoto Filho PT, Fragoso G, Sciutto E, Fleury A. Inflammation in neurocysticercosis: clinical relevance and impact on treatment decisions. Expert Rev Anti Infect Ther. 2021;19(12):1503–18. doi: 10.1080/14787210.2021.1912592 33794119

[7] Dixon HBF, Lipscomb FM. Cysticercosis: an analysis and follow-up of 450 cases. London: Medical Research Council; 1961.

[8] Chitsulo L, Engels D, Montresor A, Savioli L. The global status of schistosomiasis and its control. Acta Trop. 2000;77(1):41–51. doi: 10.1016/s0001-706x(00)00122-4 10996119 PMC5633072

[9] Donadeu M, Bote K, Gasimov E, Kim SH, Lin Z, Lucianez A, et al. WHO *Taenia solium* endemicity map – 2022 update. Wkly Epidemiol Rec. 2022;97(17):169–72. https://www.who.int/publications/i/item/who-wer9717-169-172

[10] Haby MM, Sosa Leon LA, Luciañez A, Nicholls RS, Reveiz L, Donadeu M. Systematic review of the effectiveness of selected drugs for preventive chemotherapy for *Taenia solium* taeniasis. PLoS Negl Trop Dis. 2020;14(1):e0007873. doi: 10.1371/journal.pntd.0007873 31945055 PMC6964831

[11] Kwon C-S, Jacoby A, Ali A, Austin J, Birbeck GL, Braga P, et al. Systematic review of frequency of felt and enacted stigma in epilepsy and determining factors and attitudes toward persons living with epilepsy-Report from the International League Against Epilepsy Task Force on Stigma in Epilepsy. Epilepsia. 2022;63(3):573–97. doi: 10.1111/epi.17135 34985782

[12] Mayor R, Gunn S, Reuber M, Simpson J. Experiences of stigma in people with epilepsy: a meta-synthesis of qualitative evidence. Seizure. 2022;94:142–60. doi: 10.1016/j.seizure.2021.11.021 34915348

[13] World health Organization. WHO guideline on control and elimination of human schistosomiasis. Geneva: 2022. https://www.who.int/publications/i/item/9789240041608 35235279

[14] World Health Organization. Safety in administering medicines for neglected tropical diseases. Geneva: World Health Organization; 2021. https://www.who.int/publications/i/item/9789240024144

[15] Ramiandrasoa NS, Ravoniarimbinina P, Solofoniaina AR, Andrianjafy Rakotomanga IP, Andrianarisoa SH, Molia S, et al. Impact of a 3-year mass drug administration pilot project for taeniasis control in Madagascar. PLoS Negl Trop Dis. 2020;14(9):e0008653. Epub 2020/09/19. doi: 10.1371/journal.pntd.0008653 ; PMCID: PMCPMC7500903 32946447 PMC7500903

[16] World Health Organization. Symptoms and signs compatible with neurocysticercosis in relation to preventive chemotherapy. 2023. p. 3. Available from: https://www.who.int/publications/i/item/9789240068117.

[17] World Health Organization. Symptoms and signs compatible or associated with neurocysticercosis - Poster. 2023. https://www.who.int/publications/m/item/WHO-UCN-NTD-VVE-2022.1

[18] World Health Organization. In areas where cysticercosis might be present - Poster. 2023. https://www.who.int/publications/m/item/WHO-UCN-NTD-VVE-2022.2

[19] World Health Organization. Early detection and management of neurological serious adverse events in relation to the administration of anthelminthic medicines to people with asymptomatic neurocysticercosis. 2023. p. 8. Available from: https://www.who.int/publications/i/item/9789240068131.

[20] World Health Organization. mhGAP interventions guide for mental, neurological and substance use disorders in non-specialized health settings: mental health Gap Action Programme (mhGAP) - version 2.0. Geneva, Switzerland: World Health Organization; 2016. 27786430

[21] Zafindraibe NJ, Ralalarinivo J, Rakotoniaina AI, Maeder MN, Andrianarivelo MR, Contamin B, et al. Seroprevalence of cysticercosis and associated risk factors in a group of patients examined at the Regional Referral Hospital in Antsirabe. Pan Afr Med J. 2017;28:260. doi: 10.11604/pamj.2017.28.260.10463 Epub 2018/06/09. ; PMCID: PMCPMC5989193 29881503 PMC5989193

[22] Rasamoelina-Andriamanivo H, Porphyre V, Jambou R. Control of cysticercosis in Madagascar: beware of the pitfalls. Trends Parasitol. 2013;29(11):538–47. doi: 10.1016/j.pt.2013.09.002 24145061

[23] Mananjara DEA, Rakotoarinoro M, Rakotoarison VC, Raliniaina M, Razafindraibe NP, Ravonirina C, et al. Confirmation by necropsy of a high prevalence of porcine cysticercosis in a rural district of Madagascar. Parasitology. 2023;150(9):852–7. doi: 10.1017/S0031182023000653 PMCID: PMCPMC10478050 37496390 PMC10478050

[24] Gemmell MA, Matyas Z, Pawlowski ZS, Soulsby EJL. FAO/UNEP/WHO Guidelines for surveillance, prevention and control of taeniasis/cysticercosis. Geneva: World Health Organization; 1983.

[25] Garcia HH, Gonzalez AE, Tsang VC, O’Neal SE, Llanos-Zavalaga F, Gonzalvez G, et al. Elimination of *Taenia solium* transmission in northern Peru. N Engl J Med. 2016;374(24):2335–44. doi: 10.1056/NEJMoa1515520 ; 27305193 PMC4962610

[26] Hamamoto Filho PT, Singh G, Winkler AS, Carpio A, Fleury A. Could differences in infection pressure be involved in cysticercosis heterogeneity? Trends Parasitol. 2020;36(10):826–34. doi: 10.1016/j.pt.2020.07.003 32819826

[27] Prasad KN, Verma A, Srivastava S, Gupta RK, Pandey CM, Paliwal VK. An epidemiological study of asymptomatic neurocysticercosis in a pig farming community in northern India. Trans R Soc Trop Med Hyg. 2011;105(9):531–6. doi: 10.1016/j.trstmh.2011.06.001 21764415

[28] Ronen JA, Ammu A, Gadiraju G, Vaikuntam A. An asymptomatic case of radiologically active neurocysticercosis. Cureus. 2020;12(5):e8219. doi: 10.7759/cureus.8219 Epub 2020/06/26. PMCID: PMCPMC7306638 32582480 PMC7306638

[29] Frey LC. Epidemiology of posttraumatic epilepsy: a critical review. Epilepsia. 2003;44(s10):11–7. Epub 2003/09/27. doi: 10.1046/j.1528-1157.44.s10.4.x 14511389

[30] Sáenz B, Ruíz-Garcia M, Jiménez E, Hernández-Aguilar J, Suastegui R, Larralde C, et al. Neurocysticercosis: clinical, radiologic, and inflammatory differences between children and adults. Pediatr Infect Dis J. 2006;25(9):801–3. doi: 10.1097/01.inf.0000233548.81204.97 16940837

[31] Migliani R, Rasolomaharo M, Rajaonarison P, Ravaoalimalala VE, Rabarijaona L, Andriantsimahavandy A. Cysticercosis in the port of Mahajanga: more frequent than we thought! Arch Inst Pasteur Madagascar. 2000;66(1–2):39–42. 12463033

